# Oxidative Stress and DNA Methylation in Prostate Cancer

**DOI:** 10.1155/2010/302051

**Published:** 2010-06-29

**Authors:** Krishna Vanaja Donkena, Charles Y. F. Young, Donald J. Tindall

**Affiliations:** Departments of Biochemistry/Molecular Biology and Urology, Guggenheim 501B, Mayo Clinic College of Medicine, Mayo Clinic, Rochester, MN 55905, USA

## Abstract

The protective effects of fruits, vegetables, and other foods on prostate cancer may be due to their antioxidant properties. An imbalance in the oxidative stress/antioxidant status is observed in prostate cancer patients. Genome oxidative damage in prostate cancer patients is associated with higher lipid peroxidation and lower antioxidant levels. Oxygen radicals are associated with different steps of carcinogenesis, including structural DNA damage, epigenetic changes, and protein and lipid alterations. Epigenetics affects genetic regulation, cellular differentiation, embryology, aging, cancer, and other diseases. DNA methylation is perhaps the most extensively studied epigenetic modification, which plays an important role in the regulation of gene expression and chromatin architecture, in association with histone modification and other chromatin-associated proteins. This review will provide a broad overview of the interplay of oxidative stress and DNA methylation, DNA methylation changes in regulation of gene expression, lifestyle changes for prostate cancer prevention, DNA methylation as biomarkers for prostate cancer, methods for detection of methylation, and clinical application of DNA methylation inhibitors for epigenetic therapy.

## 1. Introduction

*When diet is wrong medicine is of no use.*

*When diet is correct medicine is of no need.*

Ayurvedic Proverb [In Sanskrit, the word Ayurveda consists of the words *Ayur*, meaning “life”, and *veda*, meaning related to knowledge' or “science”].

Prostate cancer is the most commonly diagnosed cancer and a second leading cause of cancer death in men in the United States, with the vast majority of the mortality arising from the castration-resistant and/or metastatic forms of the disease [1]. Obesity and inadequate eating habits may promote prostate cancer development [2]. A healthy weight and a diet low in total fat, saturated, monounsaturated, and polyunsaturated fat and rich in omega-3 fatty acids, vitamin C, vitamin E, lycopene, alpha-tocopherol, selenium, beta carotene, and quercetin are inversely associated with prostate cancer risk [3, 4]. The beneficial effects of these nutrients in prevention of prostate cancer may be related to antioxidant levels. Among chemicals present in food, curcumin, Epigallocatechin-3-gallate (EGCG) and genistein have demethylation activity [5–7]. Epidemiological studies have indicated a link between a low occurrence of prostate cancer and diets rich in these compounds [7–9].

“Oxidative stress” is the state of a cell, which is characterized by excess production of reactive oxygen species (ROS) and/or a reduction in antioxidant defenses responsible for metabolism. ROS are formed as a natural by-product of the normal metabolism of oxygen. Under normal circumstances, the cell is able to maintain an adequate homeostasis between the formation of ROS and its removal through enzymatic pathways or via antioxidants [10]. If, however, this balance is disturbed, then oxidative stress occurs. This generates an imbalance of production/removal of ROS, which is either directly or indirectly involved in initiation, promotion, and progression phases of carcinogenesis [11]. Oxygen radicals may cause damage to DNA and chromosomes, induce epigenetic alterations, interact with oncogenes or tumor suppressor genes, and impart changes in immunological mechanisms [12, 13]. The extent of ROS-induced oxidative damage can be exacerbated by a decreased efficiency of antioxidant defense mechanisms. Endogenous defenses against ROS include antioxidant enzymes such as glutathione-s-transferase P1 (*GSTP1*), glutathione peroxidase, catalase, and superoxide dismutase [14]. Many factors such as diet, environmental carcinogens, aging, and other inflammatory diseases associated with aberrant changes in ROS may play important roles in the development and progression of prostate cancer [15–17]. Regulating such factors may offer an effective means for preventing or treating prostate cancer. 

## 2. Oxidative Stress and DNA Methylation

**Figure 1:**
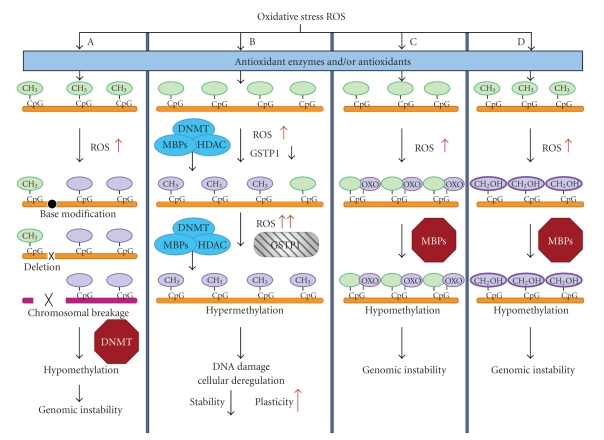
Effect of oxidative stress on DNA methylation. Antioxidant enzymes, for example, GSTP1 or antioxidants scavenge the ROS in normal cells. (A) depicts hypomethylation of DNA by ROS. 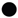, 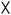, and 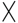 represent DNA base modification, DNA deletion, and chromosomal breakage, respectively, all of which interfere DNMT activity. (B) Under increased ROS concentrations; the MBPs, HDAC and DNMT complex methylate the CpG sites resulting in reduced *GSTP1* expression. Further increase in ROS results in complete loss of *GSTP1* (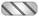) by hypermethylation. (C) and (D) represent ROS-mediated oxidation of guanine to 8-Oxy guanine and cytosine to hydroxymethyl cytosine, respectively. Both modifications interfere with MBP-mediated methylation (details are given in the text).

Oxidative stress either by metabolic, dietary, environmental, or other means leads to increased production of ROS. Generation of the hydroxyl radical can cause a wide range of DNA lesions including base modifications, deletions, strand breakage, and chromosomal rearrangements [18, 19]. Such DNA lesions have been shown to interfere with the ability of DNA to function as a substrate for the DNA methyl transferases (DNMTs), resulting in global hypomethylation (Figure 1(A)) [20]. ROS production is associated with increased DNA damage and chromosomal degradation with alterations of both hypermethylation and hypomethylation of the DNA [21]. Chronic increase of ROS in the cells can also result in lipid peroxidation and generation of a wide range of other reactive products with the potential to damage DNA [22]. Antioxidant enzymes and/or antioxidants scavenge the ROS produced in the cells. An increased vulnerability to genome-damaging stresses from electrophiles and oxidants, attributable to lack of *GSTP1*, may be the critical feature permitting prostate carcinogenesis. Inactivation of *GSTP1* may leave cells vulnerable to oxidative damage and/or tolerant to accumulation of oxidized DNA base adducts. Hypermethylation of the *GSTP1* promoter with reduced expression levels is detected in precursor high-grade intraepithelial neoplasia (HG-PIN) [23]. Absence of *GSTP1* expression with promoter hypermethylation is evident in prostate cancer (Figure 1(B)) [24, 25]. In CpG dinucleotides, the cytosine is the preferred base for DNA methylation, whereas the guanine is the site for oxidative damage. The guanine oxidative product, 8-oxoguanine (8-oxoG), is a major form of DNA damage [26–28]. Thus, it is widely used as a biomarker of oxidative damage [29]. The N7 position of guanine acts as a hydrogen bond acceptor in the formation of the methyl binding protein (MBP)-DNA complex. The oxidation of guanine to 8-oxoG converts the N7 position of guanine from a hydrogen bond acceptor into a hydrogen bond donor, as well as replaces the 8-proton with an oxygen atom. Replacement of guanine to 8-oxoG substantially diminishes MBP binding when 8-oxoG is adjacent to the 5-methyl-cytosine (Figure 1(C)) [30–33]. In addition, the methyl group of 5-methyl-cytosine is susceptible to oxidation and can generate 5-hydroxymethyl cytosine [34]. Methyl group of 5-methyl cytosine is important for sequence-specific DNA-protein interactions [31, 35]. Replacement of 5-methyl-cytosine to hydroxymethyl cytosine reverses the binding affinity to MBPs, interfering with subsequent steps in the chromatin condensation cascade, resulting in potentially heritable epigenetic alterations (Figure 1(D)).

## 3. Regulation of Gene Expression by DNA Methylation

In mammalian cells, most of the chromatin exists in a condensed, transcriptionally silent form called heterochromatin. Euchromatin is less condensed, and contains most of the actively transcribed genes. Histones and DNA are chemically modified with epigenetic markers that influence chromatin structure by altering the electrostatic nature of the chromatin or by altering the affinity of chromatin-binding proteins. DNA methylation is usually associated with histone deacetylation, chromatin condensation, and gene silencing [36–38]. DNA methylation leads to gene silencing either by inhibiting the access of target binding sites to the transcriptional activators [39] or by promoting the binding of methyl-binding domain proteins, which can mediate repression through interaction with histone deacetylases (HDACs) [40, 41] that promote chromatin condensation into transcriptionally repressive conformations. 

DNA methylation involves the addition of a methyl group to the fifth carbon position of the cytosine pyrimidine ring via a methyltransferase. This covalent modification of multiple sites on DNA by methylation is a heritable and reversible epigenetic process, which is involved in regulation of a diverse range of biological processes [42–44]. The *de novo *methyltransferases *DNMT3A* and *DNMT3B* methylate the genome during embryonic development, whereas the maintenance DNA methyltransferase *DNMT1* methylates hemimethylated DNA following DNA replication. The preponderance of DNA methylation occurs at 5′…CpG…3′ dinucleotides, but other methylation patterns do exist. In fact, 80 percent of all 5′…CpG…3′ dinucleotides are methylated, whereas the majority of the 20% that remain nonmethylated are within promoters or in the first exons of genes [45]. CpG dinucleotides are relatively infrequent in the human genome, except in CpG islands, which are (0.2 to 2 kb) regions highly enriched in CpGs [46]. Approximately 50% to 60% of gene promoters lie within CpG islands. CpG methylation outside of CpG islands is thought to suppress transcription of transposable elements and spurious initiation of transcription elsewhere. 

DNA methylation abnormalities, either gain of methylation in normally unmethylated promoters or other regulatory regions (hypermethylation), contribute to tumorigenesis by decreasing activity of tumor suppressor genes. Loss of methylation in normally methylated repetitive sequences (hypomethylation) that leads to activation of protooncogenes and genomic instability is evident in almost all human tumor types [42, 47, 48]. DNA methylation is the best-established epigenetic mark that is critical for the allele-specific expression of imprinted genes [49]. Hypomethylation of specific chromosomal domains has been linked to chromosomal instability [50]. Chromosomal abnormalities associated with hypomethylation include isochromosomes, unbalanced juxtacentromeric translocations, and whole-arm deletions. DNA hypomethylation of repetitive elements, retrotransposons, and CpG poor promoter regions plays an important role in tumorigenesis [51]. Hypomethylation of repetitive sequences and retrotransposons is associated with chromosomal rearrangements and translocation to other genomic regions, thereby promoting genomic instability [44, 52, 53]. 

## 4. Lifestyle Changes and Prostate Cancer

*The doctor of the future will give no medication, but will interest his patients in the care of the human frame, diet and in the cause and prevention of disease. * ~Thomas Edison. 

### 4.1. Dietary Factors

Epigenetic changes can be modulated by molecules that are part of our daily diet. Caloric restriction is associated with myriad changes, including an increased life span, at least in animal models, and potentially delays a wide range of diseases including cancer [54]. Increasing evidence from epidemiology and laboratory studies suggests that diet and lifestyle may have a role in the development of prostate cancer [55–57]. In a recent Prostate Cancer Lifestyle Trial (PCLT) [58], 93 men with early prostate cancer (who had opted for active surveillance before the study) were randomly assigned to either a 1-year intensive lifestyle change program or to a usual care control group [55–60]. The intensive lifestyle program included a vegan diet (supplemented with soy, fish oil, vitamin E, selenium, and vitamin C), moderate aerobic exercise (walking 30 minutes 6 days weekly), stress management techniques (gentle yoga-based stretching, breathing, meditation, imagery, and progressive relaxation for a total of 60 minutes daily), and participation in a 1-hour weekly support group to enhance adherence to the intervention [57]. The diet was predominantly fruits, vegetables, whole grains (complex carbohydrates), legumes, and soy products, was low in simple carbohydrates, and included approximately 10% of calories from fat [61]. This study found that the patients in the experimental group had a significant reduction in PSA levels and had fewer prostate cancer-related clinical events compared with the controls at the end of the 1-year program. Also, after 1 year, the growth of prostate cancer cell line, LNCaP cells was inhibited almost 8 times more by serum from the experimental than from the control group (70% versus 9%) [55]. Furthermore, the experimental patients had greater improvements in cardiovascular health parameters than did control patients, as shown by lowered total and low-density lipoprotein and cholesterol levels, which might translate into a reduction in cardiac events over the long term. This is especially important because, in general, men with prostate cancer are more likely to die of cardiovascular disease than of prostate cancer [62]. The preventive effects of this trial may be due to the reduction of stress levels and the protective effects of antioxidants from the vegetables and fruits. Many men are making changes in diet and lifestyle in the hope of preventing or slowing the progression of prostate cancer [57]. 

The anticancer properties attributed to several bioactive food components, encompassing both essential nutrients and nonessential components, may relate to DNA methylation patterns [63]. Global DNA methylation alterations in prostate cancer are correlated with adaptive changes in several signaling pathways that may be influenced by lifestyle changes. Dietary factors may influence the supply of methyl groups available for the formation of S-adenosylmethionine (SAM), a coenzyme involved in methyl group transfer. Moreover, dietary factors may modify the utilization of methyl groups by processes including shifts in *DNMT1* activities. Finally, DNA methylation patterns may influence the response to a bioactive food component. Several lines of evidence suggest that DNA hypomethylation and chromosome instability may result from insufficient dietary folate. Folate provides carbon units for a number of biochemical processes, including production of SAM, a universal methyl donor that also supplies the methyl group on cytosines in DNA. The effect of reduced dietary folate on hypomethylation is observed in dietary studies in humans, and the hypomethylation is reversible by controlled folate repletion [64]. SAM is required for the biosynthesis of the polyamines spermidine and spermine, which are produced by normal prostate secretory cells. One of the possible explanations for a limitation in SAM is the increased requirement for folate biosynthesis in proliferating cancer cells. Insufficient concentrations of SAM for DNA methylation in cancers may be caused by an insufficient supply of metabolic precursors, for example, methionine, folate, vitamin B12, zinc and choline, or increased demands from various other methylation reactions [65–67]. Methionine deprivation stress induces apoptosis, which is mediated by downregulation of *TP53* and increased production of *TRAIL* and proinflammatory cytokines [68]. Imbalances of nutrients and other bioactive food components have been shown to lead to global DNA hypomethylation, and gene-specific hypomethylation and/or hypermethylation.

### 4.2. Risk Factors

A few well-established risk factors for prostate cancer incidence include increasing age, race, ethnicity, and a positive family history. Higher ROS-mediated oxidative stress was detected more in the epithelium of prostate cancer patients than men without the disease [12]. The association of ROS with race remains to be elucidated. A racial difference in the methylation status of the *GSTP1, CD44, ESR,* and *CDH1* genes is associated with prostate cancer. A 1.7-fold higher frequency of *CD44* methylation was observed among African Americans (43%) relative to Caucasians (25%) [69]. Cigarette smoke is potentially capable of generating a high load of free radicals in the body. The effect of dietary and environmental risk factors on prostate cancer was evaluated in a recent NIH-AARP Diet and Health study. The data confirmed a number of observational studies linking smoking to prostate cancer mortality [70]. Interestingly, current (but not former) smokers had a higher mortality from prostate cancer, suggesting that smoking cessation could lead to improved survival. A significant correlation of methylation status of multiple genes with smoking status in prostate cancer has been observed [71]. Epigenetic alterations are also attractive targets of environmental carcinogenesis. Nickel and arsenic metals, butyrate: a short chain fatty acid, Phenobarbital: the tumor promoting agent, nicotine-derived nitrosamine ketone (NNK): a tobacco-specific carcinogen and methylene chloride: an occupational carcinogen, methionine and cytidine analogs are some of the agents known to alter cytosine methylation patterns of the promoter tumor suppressor genes and oncogenes [72–77].

### 4.3. Aging

*The concept that environment might change your hereditary without changing a gene sequence is the front lines of Epigenetics. As life is changing all the time, the epigenetic code that controls the DNA is turning out to be the mechanism through which we change along with it.*

Prostate cancer is mostly a disease of elderly men. The progressive inherent or acquired changes in cellular metabolism occurring with aging may play an important role in the development of this disease. ROS generated either endogenously (mitochondria, metabolic process, inflammation, etc.) or from external sources, due to decreases in intracellular ROS scavenging system plays a vital role in regulating several biological phenomena [78, 79]. There is a growing evidence that the epigenetics of an individual changes with aging, especially the accumulation of DNA methylation and histone deacetylation [69, 80–82]. Aging of the immune system, or immunosenescence, is characterized by a decline of both T and B cell function, and paradoxically the presence of low-grade inflammation. Androgen receptor (AR) is up-regulated in an age-associated manner in man and promotes continued proliferation and differentiation of the prostate [83]. Normal androgen levels can promote the production and accumulation of ROS in prostate cancer cells. Androgen-induced increase in ROS levels in prostate epithelial cells plays a key role in prostate cancer occurrence, recurrence, and progression [84]. The involvement of oxidative stress as an early event in prostate cancer development was suggested by Miyake et al. [85] who showed that androgen suppression is capable of decreasing oxidative stress. In addition, overproduction of H_2_O_2_ plays a major role in androgen-independent cell proliferation and migration of LNCaP cells [86]. However, metastatic human prostate cancers from anorchid men express transcripts encoding androgen-synthesizing enzymes and sustain intratumoural androgens at concentrations capable of activating AR target genes and maintaining tumor cell survival [87]. 

Epigenetic mechanisms linking aging to cancer include hypermethylation of the promoter of tumor suppressor genes such as *RB1, p16* and Wnt-associated factors, aberrant DNMT activity, loss of genomic imprinting, and chromosomal translocations in hypomethylated DNA sequences [88, 89]. Serum levels of Interleukin-6 (*IL-6*), which regulates the promoter activity of *DNMT1*, increase with age [90]. Total genomic 5-methylcytosine decreases during aging and is inversely proportional to the maximum life span potential of an individual [91]. A longitudinal study of 718 elderly individuals between 55 and 92 years of age demonstrated that repetitive element methylation, particularly in ALU sequences, decreases throughout aging [92]. It has been postulated that the reduction of *DNMT1* activity with age contributes to the decrease in global DNA methylation [93]. Telomerase activity is linked to multiple developmental processes, including cell proliferation, differentiation, aging, and senescence. Telomere length and rate of telomere shorting are indicators of mitotic cell age, because telomers shorten during normal cell divisions [94]. The aspect of cellular aging that is conferred by diminished telomere maintenance appears to be an important precursor to the development of many types of cancer. Shortened telomers predict poor clinical outcomes, including increased risk of metastasis and prostate-cancer recurrence in patients undergoing radical prostatectomy [95]. Comprehensive lifestyle changes significantly increase telomerase activity and consequently increased telomere maintenance capacity in human immune-system cells [96]. Recent studies have shown that tumor telomere length and integrity can be influenced by the epigenetic status of cancer cells [97]. Methylation status of subtelomeric DNA repeats negatively correlates with telomere length and telomere recombination in cancer cell lines. Treatment of human cancer cell lines with demethylating drugs results in hypomethylation of subtelomeric repeats and increased telomere recombination, which in turn could facilitate telomere elongation [98].

## 5. DNA Methylation for Early Detection and Prediction of Metastatic Risk

In recent years, there has been an enormous effort to develop specific and sensitive biomarkers for precise and accurate screening, diagnosis, prognosis, and monitoring of high risk cancer. The cancer epigenome is characterized by global changes in DNA methylation and histone modification patterns as well as altered expression profiles of chromatin modifying enzymes. Indeed, DNA methylation changes appear to be more frequent events than genetic mutations [99, 100]. If aberrant methylation of CpG sites in noncancer tissues is associated with a risk for cancer development, it may be used as a cancer risk marker. Aberrant DNA methylation may be among the earliest changes to occur during oncogenesis [101]. Once epigenetic modifications are established in premalignant tissues, the extent of modifications may accumulate as the disease progresses [102–104]. Aberrant DNA methylation of CpG sites in cancer cells may be used to detect cancer cells in biopsy samples or cancer-derived DNA in plasma. When imbalances in methylation contribute to tumor progression, methylation changes should increase in frequency and/or severity coordinately with increasing malignancy grades [24, 105]. If methylation of CpG sites is associated with a disease phenotype, then it can be used as a marker to predict phenotype, which may facilitate prognosis or prediction of responses to therapy. 

Evidence for DNA methylation as an early event comes from studies of clinical samples, where DNA methylation changes were detected in early preneoplastic lesions [106]. Of all the genes known to be methylated in prostate cancer, *GSTP1* is the most frequently methylated gene. *GSTP1 *is a detoxifying enzyme that helps to catalyze conjugation reactions between potentially damaging oxidants, electrophiles, and glutathione [107, 108]. Expression of *GSTP1 *is diminished or absent in prostate cancer, and this absence is tightly regulated by hypermethylation of the promoter CpG Island [24]. Although hypermethylation of *GSTP1* is rarely detected in normal prostate or benign prostatic hyperplasia (BPH), it is hypermethylated in >90% of cancers and about 70% of precursor high grade intraepithelial neoplasia (PIN) lesions [109, 110]. Thus, *GSTP1* methylation has improved the standard histological diagnosis in sextant biopsies [111]. In addition, *GSTP1* methylation is correlated with Gleason grade and prostate cancer volume, suggesting that quantitative *GSTP1* methylation may be of prognostic significance [112]. *GSTP1 *methylation is evident in 90% of lymph nodes from prostate cancer patients but in only 11.1% of lymph nodes from noncancer patients, suggesting that detection of *GSTP1* could have a role in molecular staging of prostate cancer [113]. The inactivation of *GSTP1* may leave cells vulnerable to oxidative DNA damage and/or tolerant to accumulation of oxidized DNA base adducts. Taken together, these results suggest that prostatic cells in proliferative inflammatory atrophy lesions, which are exposed to inflammatory oxidants, induce *GSTP1* expression as a defense against oxidative genomic damage. Cells with a defective *GSTP1* gene may become vulnerable to oxidants and electrophiles that can inflict genomic damage, which in turn may promote transformation of PIN to prostate cancer [114]. 

Analysis of multiple gene methylation patterns, as compared to that of a single gene, can improve the ability to distinguish cancerous from benign prostate tissues, and also improves correlations with pathological features such as, stage, grade, and recurrence [115]. Hypermethylation of multiple genes (including *GSTP1*, *RAR*-2*β*, and *APC)* identified prostate cancer in histopathologically negative biopsy samples collected from men who were later positively diagnosed during a follow-up biopsy procedure [116]. We have shown that hypermethylation of *RAR*-2*β*, * GSTP1, PDLIM4,* and *FLNC* facilitates the diagnosis of prostate cancer with a sensitivity and specificity of 87.3% and 87.1%, respectively [24, 82, 117]. Methylation of the *RAR-2*β** promoter could discriminate between neoplastic and nonneoplastic tissues with 94.9% sensitivity and 100% specificity [118]. Hypermethylation of a combination of genes including *APC, RASSF1A, PTGS2, PDLIM4, *and *MDR1* could distinguish cancer from benign prostate tissues with sensitivities of 97.3%–100% and specificities of 92%–100% [24, 119]. The increase in methylation of these genes with cancer progression indicates that they could be used for biomarkers for both diagnosis and risk assessment [120, 121]. Furthermore, we showed significant differences in the frequency of methylation at individual CpG sites of *PITX2, PDLIM4, KCNMA1, GSTP1, FLNC, EFS,* and *ECRG4* in recurrent and nonrecurrent subtypes of prostate tumors [24]. Indeed, hypermethylation of a CpG island in *PITX2* portended an increased risk of prostate cancer recurrence [105] and was a predictor of distant disease recurrence in tamoxifen-treated, node-negative breast cancer patients [122]. Moreover, specific CpG sites of *FLNC* and *EFS,* genes involved in cell attachment, are associated with systemic recurrence [24]. Remarkably, the combination of methylation score with GPSM score improved the theoretical prediction of recurrence. A GPSM score is a prognostic model using the weighted sum of the pathological Gleason score, preoperative PSA, seminal vesicle involvement, and marginal status to predict biochemical progression after radical prostatectomy [82]. These data suggest that DNA methylation analysis could augment the ability of currently available predictors of prostate cancer progression. 

CpG island methylation may precede genetic instability in cancer cells. The *MLH1* and *14-3-3 sigma *genes, both important for genome integrity, are frequently silenced by aberrant methylation [123]. *MLH1* encodes a DNA mismatch repair protein. *MLH1* promoter methylation and gene silencing are significantly correlated with microsatellite instability [124, 125]. Experimental demethylation in tumor cell lines results in reexpression of *MLH1* and restoration of a DNA mismatch repair proficient phenotype [126]. Hypermethylation of *hMLH1 *and *p14/INK4a *CpG islands is rare in primary cancers and more common in metastatic disease [127, 128]. DNA methylation-induced silencing of genes may be involved in the regulation of the self-renewal capacity of stem-precursor cells. For example, hypermethylation of *p16* and *APC* is commonly observed in the early stages of prostate cancer [129]. Also, other hypermethylated genes, including *CDH1, CDKN2A, CD44, CAV1, HOXD3, *and *BMP7,* have been demonstrated in prostate cancer [130–132]. Methylation of *CDH1* and *CD44* is increased in advanced prostate tumors, suggesting that they might be useful markers to assess tumor progression [131]. Comparison of methylation patterns in low and high-grade cancers suggests that *HOXD3, BMP7, *and *EDNRB* may play a role in the development of high-grade tumors [133]. Hypermethylation of *APC* and *RUNX3 *was associated with increased risk of prostate cancer-specific mortality [134].

In contrast to hypermethylation, hypomethylation of genomic 5meCytosine content in LINE1 elements and CpG islands of gene promoters may lead to overexpression of genes [135]. LINE1 elements are the largest class of repetitive elements in the human genome. Hypomethylation of LINE1 elements can lead to transcriptional activation, induction of retrotransposition, and facilitation of genetic instability [136]. There appears to be a causal relationship between hypomethylation and chromosomal instability [137]. DNA hypomethylation occurs late in prostate cancer progression and is likely to be involved in the formation and progression of metastases [135]. DNA hypomethylation is a significant source of tumor heterogeneity in metastatic prostate cancer and may contribute to the development of therapeutic resistance [138, 139]. Gene-specific hypomethylation can cause heterogeneous overexpression of a series of cancer-testis antigen genes (CTA), many of which are currently being evaluated as targets of immunotherapy. Clinical trials have shown regression of tumors when patients are treated with immunotherapies targeted to these CTA antigens [140, 141].

Noninvasive and minimally invasive tests, particularly those that provide molecular signatures in blood samples, may enhance our ability to detect prostate cancer [142, 143]. Cell-free circulating DNA in blood plasma exhibits cancer-associated changes in DNA methylation, and thus represents an attractive biomarker assay. Hypermethylation of *GSTP1 *was found in 94% of tumors, 72% of plasma or serum samples, 50% of ejaculate, and 36% of urine from patients with prostate cancer [144, 145]. Hypermethylation of *GSTP1* CpG island sequences could be detected in prostatic secretions collected from 96% of radical prostatectomy specimens [146]. The abnormal DNA methylation patterns in these secretions may have come from prostate cancer cells, or from PIN cells shed into prostate ducts. Methylated DNA in blood and urine may serve as a screen for prostate cancer and may identify men at risk for developing aggressive disease. Indeed, a dual-assay based on both genetic and epigenetic alterations in multiple microsatellite and methylation markers in circulating DNA from serum samples exhibited greater sensitivity for prostate cancer detection than that of a single-marker assay and was independent of PSA levels or the American Joint Cancer Committee (AJCC) stage [147]. Prognostic markers may help to identify those patients who will recur with cancer. Furthermore, accurate risk prediction may help identify patients who would benefit from more aggressive treatments immediately following primary therapy or select patients for active surveillance.

## 6. Methods for Detection of DNA Methylation

Epigenome mapping is inherently complex, since the epigenome varies with age, differs between tissues, is altered by environmental factors, and shows aberrations in disease. In an era of synthetic genomics and personalized medicine, mapping of the epigenome at different ages, in different tissue types and disease states should shed light on novel biological functions and phenotypic differences of heterogeneous prostate cancer. The ability to detect and quantify DNA methylation efficiently and accurately is important for prostate cancer diagnosis. High resolution analysis of individual CpG sites involves the chemical modification of DNA by bisulfite treatment, where sodium bisulfite deaminates cytosine into uracil, whereas methylated cytosine is resistant to this conversion. Measurement of methylation levels involves bisulfite conversion, followed by real-time PCR [82, 148], base-specific cleavage and mass spectrometry [24, 149], Pyrosequencing [150], combined with bisulfite restriction analysis (COBRA) [151] or methylation-sensitive single nucleotide primer extension (Mu-SNuPE). The limitation of these methods is the cost and scalability. 

To comprehensively characterize the molecular effects of DNA methyltransferase inhibitors, high-resolution methods need to be developed to analyze genome-wide methylation patterns. These methods can also be used to develop and refine epigenetic therapies for cancer. If such methods can be established, they will allow direct comparison of the biologic effectiveness of demethylation agents, as well as the optimization of schedules and the rational designs of combined treatments with DNA methylation inhibitors and other anticancer drugs. Genome-wide approaches to analyze methylation involve comparative microarray hybridization following fractionation of the genome, based upon methyl-cytosine-specific antibodies and protein complexes or methylation-specific enzymes with sites in CpG-rich regions [152–154]. The sensitivity of the enzymatic approach is limited by the sequence context of the digestion site and by the number of sites available. Bisulfite sequencing represents the most comprehensive, high-resolution method for determining DNA methylation states. Accurate quantification of variable methylation frequencies requires high sampling of individual molecules. High-throughput, single-molecule sequencing instruments have facilitated the genome-wide application of this approach. However, these approaches are cost ineffective and currently are impractical for routine application in complex genomes with many epigenomic states. Recent strategies for addressing methylation in large genomes include enzyme directed reduced genomic representation followed by parallel sequencing [155, 156] and bisulfite capture technology, which combines bisulfite conversion with hybrid selection techniques and deep sequencing [157]. Bisulfite capture directs focus to specified CpG regions in a highly parallelized process designed to selectively enhance sequence information content by deeper sampling of targeted bases. In addition, most of these techniques are highly labor intensive and cannot be automated. Nanotechnology platforms based on nanopore or nanowire-transition based ultra sensitive detection of the methylated DNA show promise for routine clinical diagnostics in the future [158, 159]. 

## 7. Epigenetic Therapy

Epigenetic changes are reversible, raising the possibility of epigenetic therapy, which has led to the development of epigenetic anticancer drugs such as demethylation agents and histone deacetylation inhibitors (HDAC-I). Many genes encoding enzymes, drug transporters, transcription factors, cell cycle regulators, and nuclear receptors are under epigenetic control. Thus, pharmacoepigenetics offers a potential mechanism for monitoring individual responses to treatment that cannot be accounted for solely on the basis of genetic polymorphisms. Ongoing studies to identify genes that are differentially expressed in cancer cells versus normal cells are providing valuable information about molecular targets for epigenetic therapy [160]. Some drugs that inhibit DNA methyltransferases have been shown to reactivate silenced genes and induce differentiation or apoptosis of malignant cells. 

Two inhibitors of DNA methyltransferases, 5′-azacytidine (Vidaza), and its derivative 5-aza-2′-deoxycytidine (decitabine) have already been approved by the FDA as effective drugs for treatment of myelodysplastic syndromes [161]. 5′-Azacytidine is a nucleoside inhibitor that is incorporated into DNA. DNA methyltransferase methylate both cytosine residues and 5′-azacytosine residues in the DNA. However, 5′-azacytosine prevents the resolution of a covalent reactive intermediate [162]. This leads to the DNA methyltransferase being trapped and inactivated in the form of a covalent protein-DNA adduct, which results in depletion of cellular DNA methyltransferase. 5-Azacytidine is a ribose nucleoside and thus must be chemically modified to a deoxyribonucleotide triphosphatase to be incorporated into DNA. Before 5-azacytidine is converted into deoxyribonucleoside triphosphate, a portion of it is incorporated into RNA, which affects a variety of cellular processes independent of demethylation [162]. Decitabine, the deoxyribose analogue of 5-azacytidine, exhibits more specificity with greater inhibition of DNA methylation and less toxicity than 5-azacytidine. However, it also has substantial toxic effects. Other drugs affect the epigenome, such as zebularine, which is more stable than 5-azacytidine or decitabine cytidine analog. The demethylation activity of zebularine may also be difficult to separate from the toxic effects of DNA methyltransferase depletion that results from covalent enzyme trapping [163]. 

Some nonnucleoside compounds also inhibit DNA methylation. EGCG, the main polyphenol compound in green tea, binds to and blocks the active site of *DNMT1* [7]. However, degradation of EGCG generates a substantial amount of hydrogen peroxide [164] that might contribute cytotoxic activity. RG108, a small-molecule inhibitor directly and specifically inhibits *DNMT1* with low toxicity [165]. Oligonucleotides, including hairpin-loops of DNA and a specific antisense oligonucleotide, MG98, represent another class of DNA methyltransferase inhibitors. Hairpin-loops of DNA, which are competitive substrates for DNA methyltransferases, are able to induce a weak expression of the tumor suppressor gene *p16* [166]. MG98 has exhibited antitumor activity in preclinical trials and is currently being tested in a phase II clinical trials. Psammaplins, a natural product derived from the sea sponge pseudoceratina purpurea, inhibits DNMTs as well as histone deacetylases. SAHA (suberoylanilide hydroxamic acid), an HDAC inhibitor, has been approved by FDA for the treatment of T cell cutaneous lymphoma. Several other HDAC inhibitors such as depsipeptide and phenylbutyrate are currently in clinical trials. In addition to DNA methylation and HDAC inhibitors, histone arginine methyltransferases are emerging anticancer targets, due to their role as coregulators of the androgen receptor [167]. The histone methyltransferase inhibitor DZNep induces apoptosis in cancer cells by selectively targeting polycomb repressive complex 2 (PRC2) proteins, which are generally overexpressed in cancer cells [168]. Also, combinations of DNA methylation and HDAC inhibitors with classic chemotherapeutics have shown promise in solid malignancies [169]. However, many studies suggest that demethylation of specific genes need not always result in reexpression [170, 171]. For example, demethylation of the *MAGE *gene appears to lead to reexpression only when the appropriate tissue-specific transcription factors are present [172]. Thus, various factors including nonspecific global hypomethylation and cytotoxic side effects may contribute to the complex alterations observed after epigenetic drug treatments. The characterization of these effects and development of compounds that specifically reverse abnormal DNA methylation patterns or epimutations will be required for future cancer therapies. The broad use of decitabine in cell culture experiments indicates that demethylation of the tumor suppressor genes can occur at drug concentrations lower than those required for cytotoxicity [173]. Treatment schedules have to be modified to include multiple courses of treatment to sustain demethylation and reduce drug concentrations to decrease the severity of side effects. 

As DNA methylation and hypoacetylation have been shown to contribute to silencing of chemotherapeutic sensitive genes; reversal of these modifications to allow reexpression of such genes is one possible second-line treatment for prostate cancer. These treatments would then be combined with conventional first-line therapies to elicit tumor regression. In a comprehensive study of several tumor cell lines, 5-aza-deoxycytidine allowed for apoptotic resensitization to a variety of agents, including doxorubicin and cisplatin [174]. Demethylating agents, HDAC inhibitors or combinations may allow for reexpression of silenced tumor suppressors such as *hMLH1* and *RASSF1A*. A loss of *hMLH1 *and *RASSF1A *contributes to multidrug resistance phenotype [175]. Epigenetic reexpressions of these genes might allow for resensitization of tumors to the conventional first-line therapies. Other epigenetic targets could be methyl binding proteins and miRNAs, which play a role in tumor suppressor silencing [176]. Resistance of human tumor xenografts to treatment with cisplatin, carboplatin, temozolomide and epirubicin was decreased by adding nontoxic doses of decitabine [177, 178]. Importantly the timing of drug administration appears to be associated with therapeutic responses. Structurally designed small molecule inhibitors may enhance specificity in epigenetic targeting, avoiding the potential detriments of global demethylation and hyperacetylation.

## 8. Conclusion

Chromatin structure and packaging of the genome is important for regulating the cellular homeostasis. ROS-induced oxidative stress is involved in the multistage process of prostate cancer progression. In particular, there is a growing interest in the involvement of oxidative stress in the epigenetic regulation of gene expression and specifically in controlling DNA methylation. Agents that prevent the production and chronic accumulation of ROS might play an important role in the treatment of prostate cancer. Epigenetic alterations are clearly involved in prostate cancer initiation and progression. Hypermethylated genes can be used to detect early stage of prostate cancer. In addition to the use of epigenetic alterations as a means of screening, epigenetic alteration may help clinicians to predict the risk of recurrence and drug resistance. A combinatorial approach of epigenetic therapy with antioxidant agents along with standard radiotherapy and targeted anticancer therapy may help in sensitization of tumors which are resistant to current approaches of treatment. Finally, a link between the biomarkers and therapy may have positive impact on health care.
